# Self-organized emergence of folded protein-like network structures from geometric constraints

**DOI:** 10.1371/journal.pone.0229230

**Published:** 2020-02-27

**Authors:** Nora Molkenthin, Steffen Mühle, Antonia S. J. S. Mey, Marc Timme

**Affiliations:** 1 Chair for Network Dynamics, Institute for Theoretical Physics and Center for Advancing Electronics Dresden (cfaed), Technical University of Dresden, Dresden, Germany; 2 Network Dynamics, Max Planck Institute for Dynamics and Self-Organization (MPIDS), Göttingen, Germany; 3 University of Göttingen, Third Institute of Physics – Biophysics, Göttingen, Germany; 4 EaStCHEM School of Chemistry, University of Edinburgh, Edinburgh, Scotland, United Kingdom; Weizmann Institute of Science, ISRAEL

## Abstract

The intricate three-dimensional geometries of protein tertiary structures underlie protein function and emerge through a folding process from one-dimensional chains of amino acids. The exact spatial sequence and configuration of amino acids, the biochemical environment and the temporal sequence of distinct interactions yield a complex folding process that cannot yet be easily tracked for all proteins. To gain qualitative insights into the fundamental mechanisms behind the folding dynamics and generic features of the folded structure, we propose a simple model of structure formation that takes into account only fundamental geometric constraints and otherwise assumes randomly paired connections. We find that despite its simplicity, the model results in a network ensemble consistent with key overall features of the ensemble of Protein Residue Networks we obtained from more than 1000 biological protein geometries as available through the Protein Data Base. Specifically, the distribution of the number of interaction neighbors a unit (amino acid) has, the scaling of the structure’s spatial extent with chain length, the eigenvalue spectrum and the scaling of the smallest relaxation time with chain length are all consistent between model and real proteins. These results indicate that geometric constraints alone may already account for a number of generic features of protein tertiary structures.

## I. Introduction

Proteins consist of sequences of amino acids. The resulting *primary structure* of a protein, is expected to provide constraints for the folded three-dimensional (3D) structure of a globular protein, its *tertiary structure*. The problem of predicting the 3D structure of an amino acid sequence in an aqueous solution is known as the protein folding problem consisting of three sub-problems: First, to find the chemically active folded state; second, to uncover the pathway to get to that state; and third, to develop computational tools capable of accurately predicting the folded state [1–6]. Many different avenues have been taken to explore solutions towards this problem, ranging from atomistic models using molecular dynamics approaches [7], to coarse grained models e.g [8], and to machine learning-based and heuristic physical models that disregard the atomistic details of the amino acid sequence [9, 10]. While much progress has been made improving molecular dynamics simulations using atomistic detail, the folding process of long chains is computationally highly expensive or even infeasible, and still requires access to purpose build massively parallel computers such as Anton [11], or distributed computing projects such as folding@home in order to generate quantitative data [12]. The other avenue often explored for structure models is tested in community-wide challenges such as the ‘Critical Assessment of Protein Structure Prediction’ (CASP) [13–15]. CASP is run every other year to see if a protein’s tertiary structure can be predicted based on its primary sequence of protein structures unresolved at the time of the challenge [16]. Predictions have improved drastically over previous CASP challenges [1], however, often rely on existing structural information in the protein data base (PDB) and homology modeling, comparing new proteins based on existing insights from known template proteins using computational models such as HHPred [17] or I-TASSER [18] or, more recently, machine-learning based predictions of the distance matrix [19]. These approaches support accurate prediction of 3D structures, yet by construction limit insights into fundamental physical mechanisms and constraints underlying the folding processes and final structures observed in the many and various proteins observed in nature.

In a complementary approach, a number of theories and structure analyses have been conducted into broader mechanisms of the folding process. Examples include tube models as in [20], where it was shown that secondary structures, such as helices and *β*-sheets, arise from explicit hydrogen bonds. Alternatively the behaviour of the chain can be expressed through a heuristic field equation of the backbone curvature, as in [21], where helices and sheets constitute the energy minimum. For this reason we here want to focus on the tertiary structure.

We propose an approach to further understand geometry and formation processes using a complex network framework. The 3D tertiary structures in our model arise from chain-like primary protein structures without comparing to specifically chosen protein structures available on the PDB, and without using complex molecular dynamics simulations. First, we analyze 1122 protein structures from the PDB, consider them as an ensemble of network structures representing protein tertiary structures, and quantify overall properties of this ensemble. In particular we (i) uncover the scaling of the diameter of proteins with their chain length, (ii) reveal the distribution of the number of other amino acids any given amino acid closely interacts with and (iii) find the distribution of second largest eigenvalues of their associated graph Laplacians, characterizing the most persistent time scales on which proteins are dynamically responding to perturbations. Second, we propose and analyze a simple stochastic process modeling the folding of chains of units. The minimal model takes into account geometric constraints only and does not consider any other protein property. The model process keeps connected units connected, forbids geometric overlap of units (volume exclusion) and connects randomly chosen units if geometrically permitted. Based only on such random monomer interactions and geometric constraints, akin to those in Lennard-Jones clusters and sticky hard spheres [22, 23], the 3D structures self-organizing through the simple model process are consistent with those of real protein ensembles in all of the above-mentioned features simultaneously.

These results suggest that beyond the details of pairwise interaction of amino acids, from intermediate scales of a few amino acids to the full spatial extent of proteins, geometric constraints play an important role in structure formation and strongly impact the final protein tertiary structure. Our insights may put into perspective the influence of the specific details of sequences of amino acids relative to simpler geometric constraints on structure forming processes of proteins.

## II. Methods

### A. Ensemble analysis of protein residue networks

With their modular polymer structure and their complex interaction patterns, proteins lend themselves naturally to a description as ensembles of complex networks. The mathematical object of a graph, simply termed network, represents a structure of nodes (units) and links, each describing an interaction between two units [24–26]. Networks and graphs have been used to describe the structure of a wide variety of systems, as different as social networks [27–29] and the global climate system [30, 31]. In this article, we analyze an ensemble of 1122 protein tertiary structures of chain lengths ranging from *N* = 8 to *N* = 1500 amino acids. Detailed structures have been experimentally determined to great accuracy and stored in the protein data bank (PDB) [32]. Part of the information stored in the PDB are the coordinates $x_{i}\in {\mathbb{R}}^{3}$ of the individual amino acid’s central carbon atoms *C*_*α*_, where *i* indexes the amino acid’s position along the chain.

Given such geometric data, the structures resulting from protein folding are commonly expressed as protein residue networks (PRN’s) [33–36], in which the central carbon atom of each amino acid is taken to be a node and a link represents the interaction of two nodes if their spatial distance is small, i.e. less than a distance *d*_*c*_ apart.

Here, the distance between the amino acids indexed *i* and *j* is given by the Euclidean distance metric *d*_*i*,*j*_ = ∥*x*_*i*_ − *x*_*j*_∥. An adjacency matrix *A*_*ij*_ encodes the topology of a network, its entries are 1 if *d*_*i*,*j*_ ≤ *d*_*c*_, i.e. the units are considered connected, and 0 otherwise. The distance matrix resulting from PDB data thus defines the adjacency matrix as

$$\begin{array}{c} A_{ij}^{\text{PDB}}=\{\begin{array}{cc} 0, & \;\text{if}\;d_{i,j}>d_{c}\;\text{or}\;i=j\\ 1, & \;\text{if}\;d_{i,j}\leq d_{c}. \end{array} \end{array}$$ (1)

**Fig 1:**
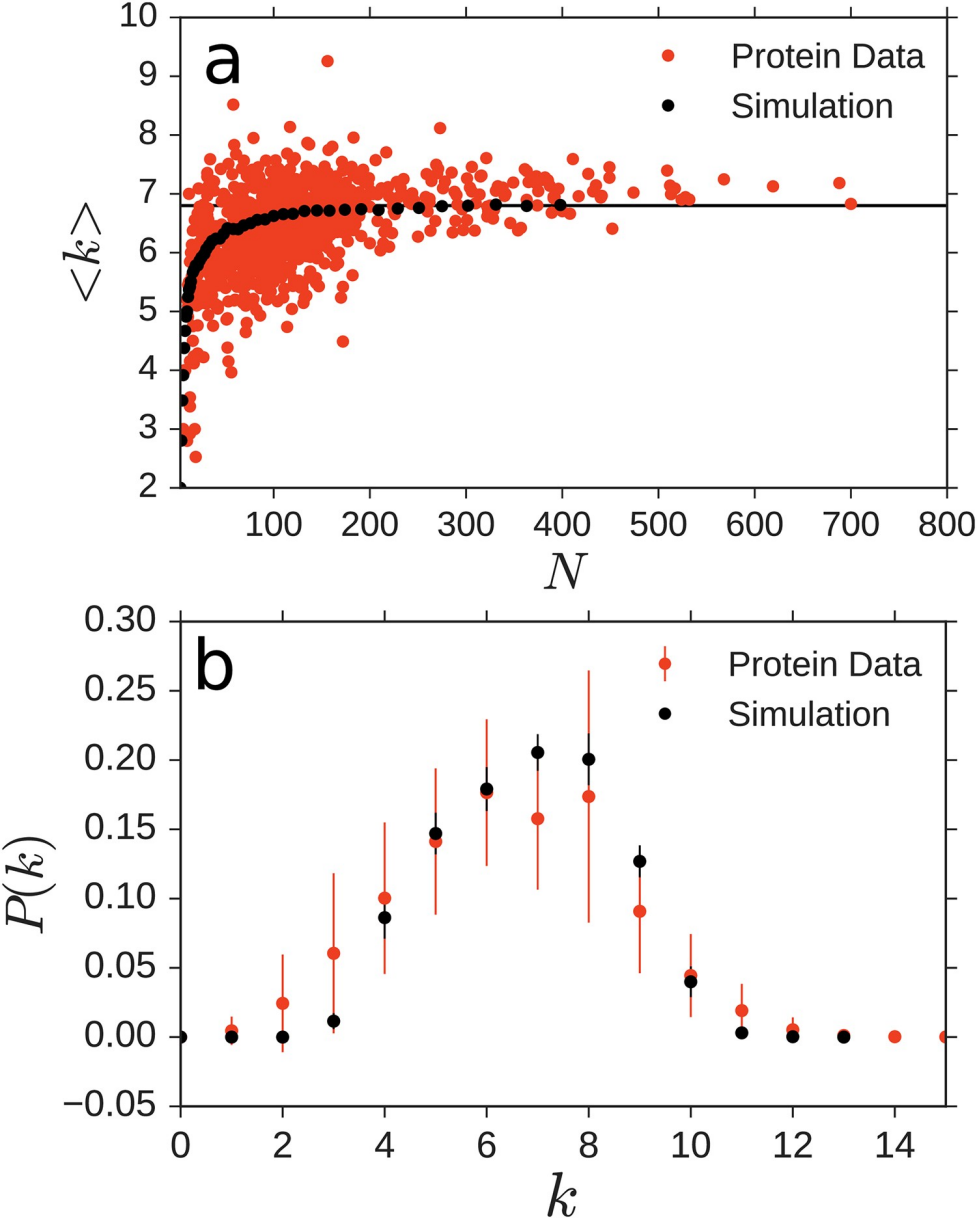
Degree distributions of simple model ensemble and real proteins are statistically indistinguishable. a) The average degree *k* of real protein ensemble (red dots) asymptotically saturates to *k* ≈ 6.8 as the chain length *N* becomes large. The average degree of the nodes resulting from 30 model simulations for each chain length *N*, ranging from *N* = 3 to *N* = 398. b) The degree distribution *P*(*k*) of the model simulation within the error margin is indistinguishable from that of real proteins (error bars indicate standard deviation of the distribution at each *k*).

The threshold of the PRN is commonly chosen between *d*_*c*_ = 4 Å(approximate length of a peptide bond [35]) and *d*_*c*_ = 8 Å, reflecting an upper bound for a significant interaction to occur between two units [35]. Here, we created the PRNs of 1122 proteins selected from the PDB list in [37], covering a range of chain lengths *N* for comparison to simulations. Their geometric structures have been determined previously via NMR and x-ray studies. We choose a threshold value of *d*_*c*_ = 6.5 Å to calibrate the average degree (the degree *k*_*i*_ of node *i* counts the number of nodes it is connected to) of nodes in the PRNs to the average degree found in the model simulations in the range of large *N* ∈ [200, 400], Fig 1a. The average degree *k* grows with *N* and appears to saturate at a value determined by *d*_*c*_. The ratio of this cutoff threshold and the unit size in the model, which we take half their mean distance, constitutes the only free structural parameter we employ in the current study.

The degree distribution of the resulting network ensemble, displayed in Fig 1b, is unimodal and covers effective degrees between *k* = 2 and *k* = 11. Interestingly, the degree distribution resulting from simulations of the model ensemble we are about to introduce below is statistically indistinguishable from those of the network of real PRNs (no additional fit parameter), Fig 1b. Equally, other quantifiers obtained from the simple, geometry-only model ensemble agree surprisingly well with those obtained from our data analysis of the experimentally obtained protein structures. For the network measures and manipulations NetworkX [38] was used.

### B. Simple model focused on geometric constraints

To better understand the impact of geometric constraints on the topology of protein tertiary structures, we introduce a random network formation model that takes into account geometric constraints and leaves out almost all other properties of real proteins, including heterogeneous sequences of amino acids, the amino acids’ specifics molecular properties, different forms of electrochemical interactions, conformational details of interactions between nearby amino-acids, and the influence of the fluid environment on protein folding. This formation mechanism can be interpreted as the intersection of random graphs and self-avoiding random walks, which has vastly different properties from the two individual sets. We find that the simple, geometry-centered model already reproduces a range of overall topological properties of real protein residue networks well.

The model is built on the simple observation that proteins consist of a chain of close-to identical units that interact in complex ways when folding, yet can not intersect, giving rise to geometric constraints. The individual units of the chain interact when they come into contact; typically there is an attraction that is the stronger the closer they are but repelling once they overlap. Depending on the specific amino acid, size, shape, and electromagnetic properties vary. In our model, however, all amino acids are represented as unit spheres and the interactions between each pair become very simple and identical across all pairs.

The model’s initial state consists of a chain of *N* connected spheres, each of diameter and bond length of unity (later rescaled to match the mean distance between neighboring amino acids *d*_mean_). A folding proceeds by sequentially picking random pairs of spheres (not connected with each other) and connecting them if possible, given the geometric constraints of volume exclusion. Here, volume exclusion also applies to co-moving other spheres connected either initially along the chain or through a previous step (see S1 File). The process repeats until all pairs are either connected or geometrically incapable of connecting. The adjacency matrix *A*^sim^ of the simulated chain keeps track of which spheres are linked to each other. Initially, it contains only zeros except for its secondary diagonal elements which equal 1 since neighboring spheres are connected via the backbone chain. The model is motivated by a two-dimensional model of network-based formation of aggregates where link constraints due to geometry in space have been approximately mapped to purely graph-theoretic constraints during network formation [39].

As described in the method section, the process of moving spheres towards each other is realized in a simple consistent way to satisfy all geometric constraints continuously in time. The forces and potentials employed, however, are *not* intended to reflect any physical forces or potentials created by amino acids. They plainly help to realize to attempt the joining of two randomly selected spheres.

**Fig 2:**
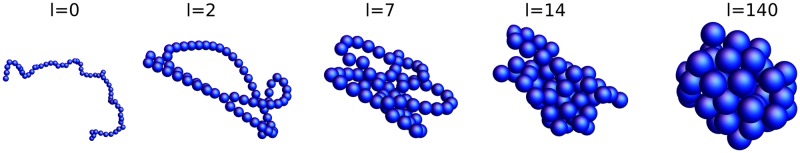
Model folding process at different times. Starting from an initial chain with *N* = 60, randomly picked units connect if geometrically possible. Shown here are examples after *l* = 0, 2, 7, 14 and 140 successful connection attempts.

**Fig 3:**
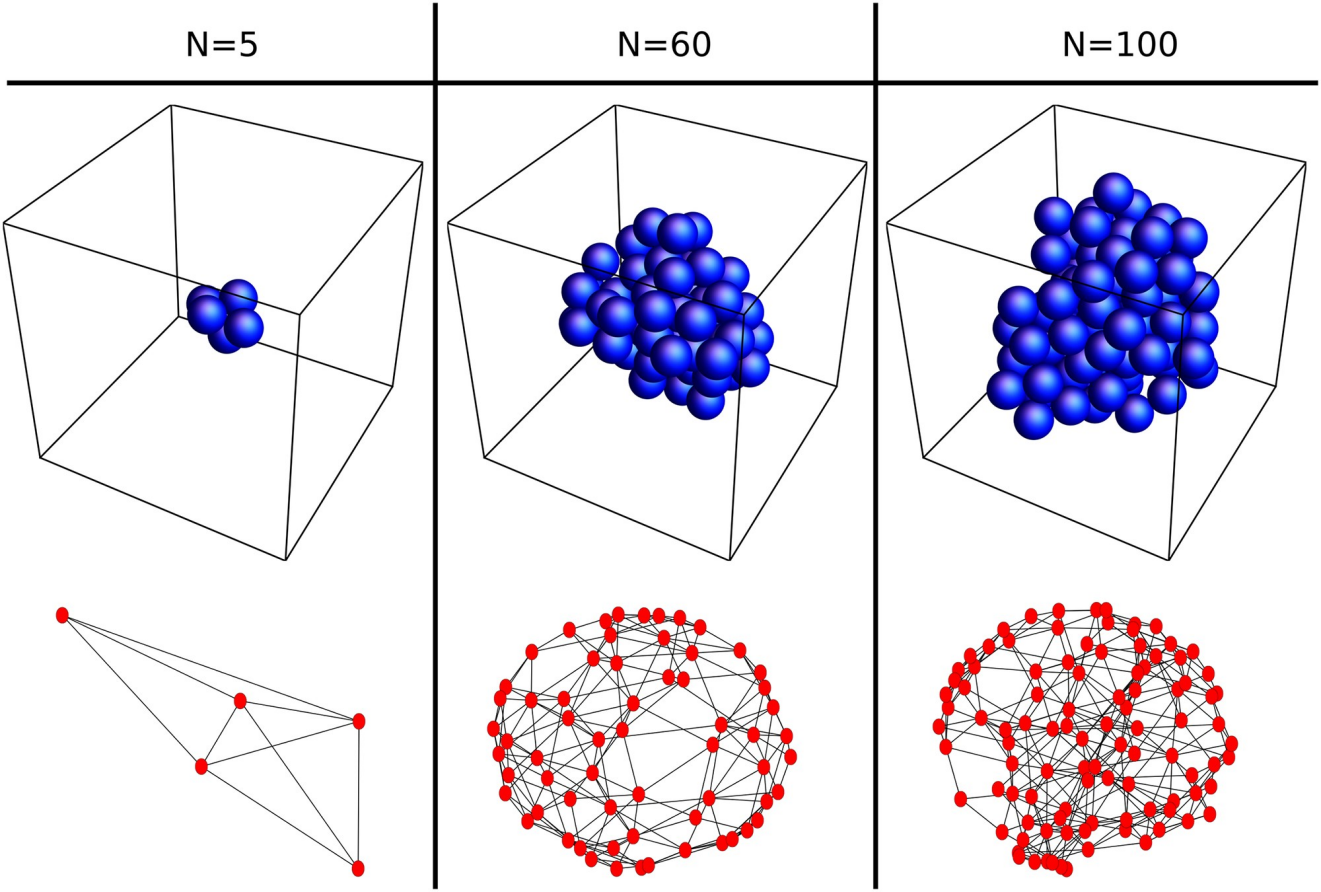
Final model aggregates. The final aggregates of the simulation for *N* = {5, 60, 100} display the expected compactness. The corresponding networks are non-planar.

Snapshots of the folding process are illustrated in Fig 2, three examples of the final aggregates in Fig 3. The aggregates are highly compact compared to the straight initial conditions. They are also much more compact than aggregates generated from self-avoiding random walks and close to, yet not quite maximally densely packed (see below), consistent with previous suggestions based on 2D aggregates [39].

All simulation details, including the code for reproducing the geometric constraint simulations, as well as the preparation and analysis of PDB files can be found in the following github repository: https://github.com/ppxasjsm/Geometric-constraints-protein-folding.

## III. Results

### A. Spatial scaling of protein structures

**Fig 4:**
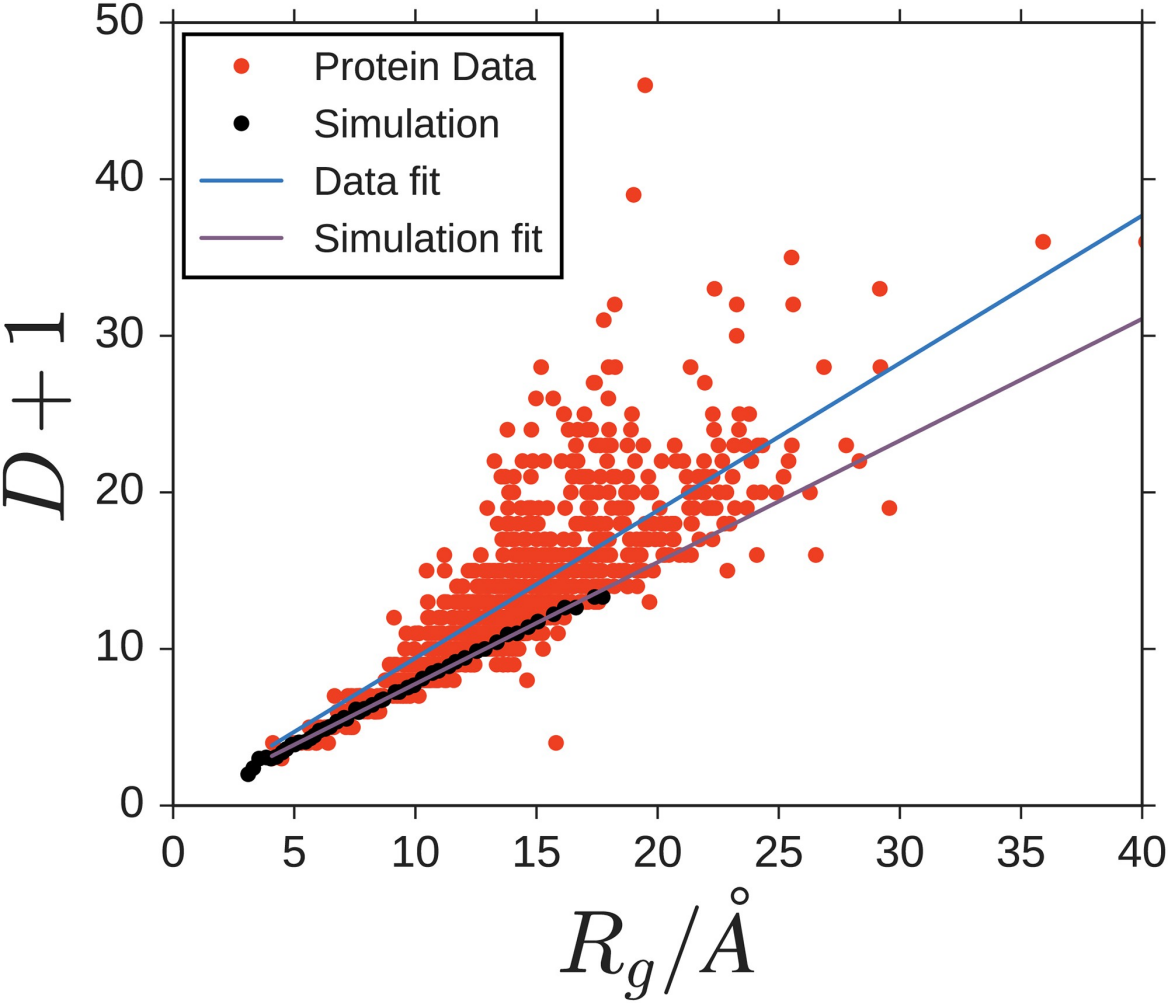
The network diameter *D* scales linearly with the radius of gyration *R*_*g*_. This holds for both biological protein residue networks and simulated model networks. Scaling the model link length to the average link length of the PRN (see text for details), yields a scaling of the graph diameter of model networks within the experimentally observed range. The best fitting proportionality constant, however, differs, with $\frac{\partial D}{\partial R_{g}}=0.942{Å}^{-1}$ for experimental data and $\frac{\partial D}{\partial R_{g}}=0.777{Å}^{-1}$ for the model data.

The ensemble of protein tertiary structures exhibits an algebraic scaling law indicating that their radii of gyration *R*_*g*_ depend on their chain length *N* such that:

$$\begin{array}{c} R_{g}∼N^{\nu }, \end{array}$$ (2)

as expected from a number of previous studies [21, 37, 39, 40]. As the overall geometry of a folded protein is often characterized by the locations of the central carbon atoms (*C*_*α*_-atoms, one for each amino acid) of its backbone chain, its spatial extension is commonly measured by the radius of gyration

$$\begin{array}{c} R_{g}={\left(N^{-1}\sum_{i}{\left(x_{i}-\bar{x}\right)}^{2}\right)}^{1/2}, \end{array}$$ (3)

quantifying the average distance of units from the center of mass $\bar{x}$, where *x*_*i*_ is the location of unit *i* ∈ {1, …, *N*}. Our previous study [39] revealed that the scaling law indeed is algebraic and that the exponent *ν* is (slightly) larger than for space filling aggregates (where $\nu_{\text{SF}}=\frac{1}{3}=0.3333\ldots$ in 3D) yet (far) smaller than for aggregates created through a self-avoiding random walk (where $\nu_{\text{RW}}=\frac{3}{5}=0.6$ in 3D). That study found *ν* = 0.3916±0.0008 for 37162 proteins. For our smaller data set of 1122 proteins, we find *ν*_*exp*_ = 0.374±0.03, see Fig 4 for illustration.

To compare the spatial extent of model aggregates, i.e. graph-theoretically defined networks of spheres, to biological proteins on the same footing, we first study how the network diameter *D* compares to the radius of gyration defined through Eq (3). The graph diameter is defined as the maximum number of links to be taken on the shortest link sequence (also referred to as shortest simple paths) between any pair of units in the PRN. We find that *D* is strongly linearly correlated with the spatial extent *R*_*g*_ of the PRN, Fig 4. Both the ensemble of biological proteins and the model ensembles studied exhibit a roughly proportional dependence of *D*+ 1 on *R*_*g*_, with the slope obtained from the model data ($\frac{\partial }{\partial R_{g}}D=0.777{Å}^{-1}$) being lower and more precisely determined than that obtained for the PRNs ($\frac{\partial }{\partial R_{g}}D=0.942{Å}^{-1}$). As proportionality factors do not affect the scaling, we thus also find

$$\begin{array}{c} \left(D+1\right)∼\;N^{\nu }, \end{array}$$ (4)

for both the PDB proteins and geometric-constraint model.

With the cutoff distance for the creation of networks chosen to be *d*_*c*_ = 6.5 Å the resulting average link length in the biological proteins becomes *d*_*mean*_ ≈ 5.066 Å, which in Fig 4 we substituted for the unit length of our model simulations. In the PRNs the network diameters are more dispersed. The lower bound of the experimental data fits well with the simulated structures, suggesting geometric constraints as a major driving mechanism influencing the spatial density during network formation.

**Fig 5:**
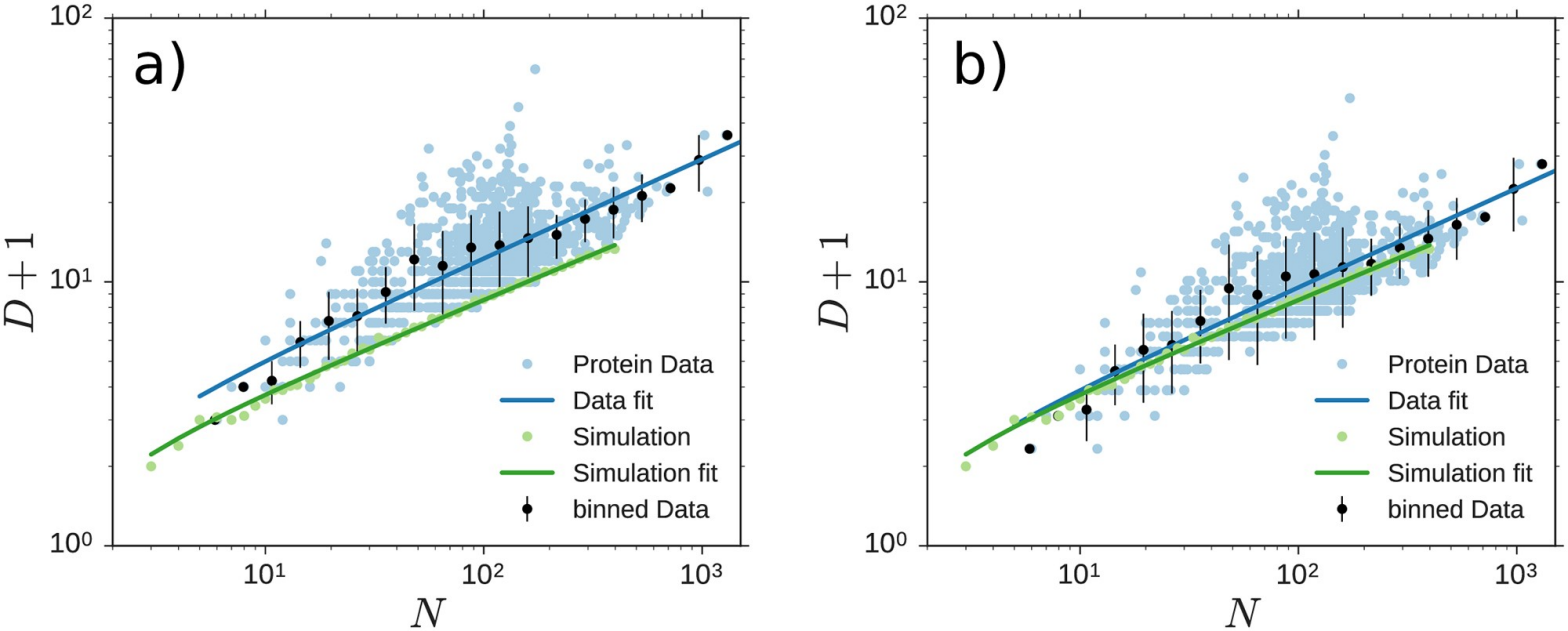
Diameter scaling with chain length. (a) The diameter *D* of simulated and measured PRN’s scales according to Eq 4 with the chain length *N*. The model results coincide with the lower bound of measured results, which we attribute to the fact that we fold maximally. (b) Matching the proportional scaling relation between graph diameter *D* and radius of gyration (Fig 4) yields scaling relations between aggregate extent and chain length to be statistically indistinguishable between model and real proteins. For both panels, we simulated 30 random dynamic realizations each for 48 aggregate lengths *N* with logarithmically spaced between *N* = 3 to *N* = 398. The data displayed shows the network diameter averaged across realizations as a function of chain length.

Both ensembles show power-law scaling of the diameter. The exponent of *ν*_*sim*_ = 0.345±0.01 of the simulation is very close to the value of *ν*_*exp*_ = 0.374±0.03, measured in the PDB data. The plots are shown in Fig 5. Simulations for heterogeneous systems where the radii of individual units are drawn randomly from the uniform distribution on [1 − *a*, 1 + *a*] for *a* ∈ {0.0, 0.1, 0.2, 0.3, 0.4, 0.5} increased the variance of the measurements for the radius of gyration, as expected. We did not observe any significant bias in the averages such that the scaling relations stay the same also for heterogeneous systems. The simulated results are found to align very well with the lower bound of folded protein diameters, suggesting that much of the discrepancy (constant factor shifting the measured results up in Fig 5) can be explained by the fact that the simulation only ceases to make new links when this is no longer geometrically possible. In real proteins on the other hand interactions range from Van-der-Waals interactions to hydrogen bonds and individual monomers vary in size and chemical properties and are subject to thermodynamic fluctuations. All this leads to larger gaps within the folded molecule and hence larger diameters of the PRN’s.

### B. Distribution and scaling of Laplacian eigenvalues

**Fig 6:**
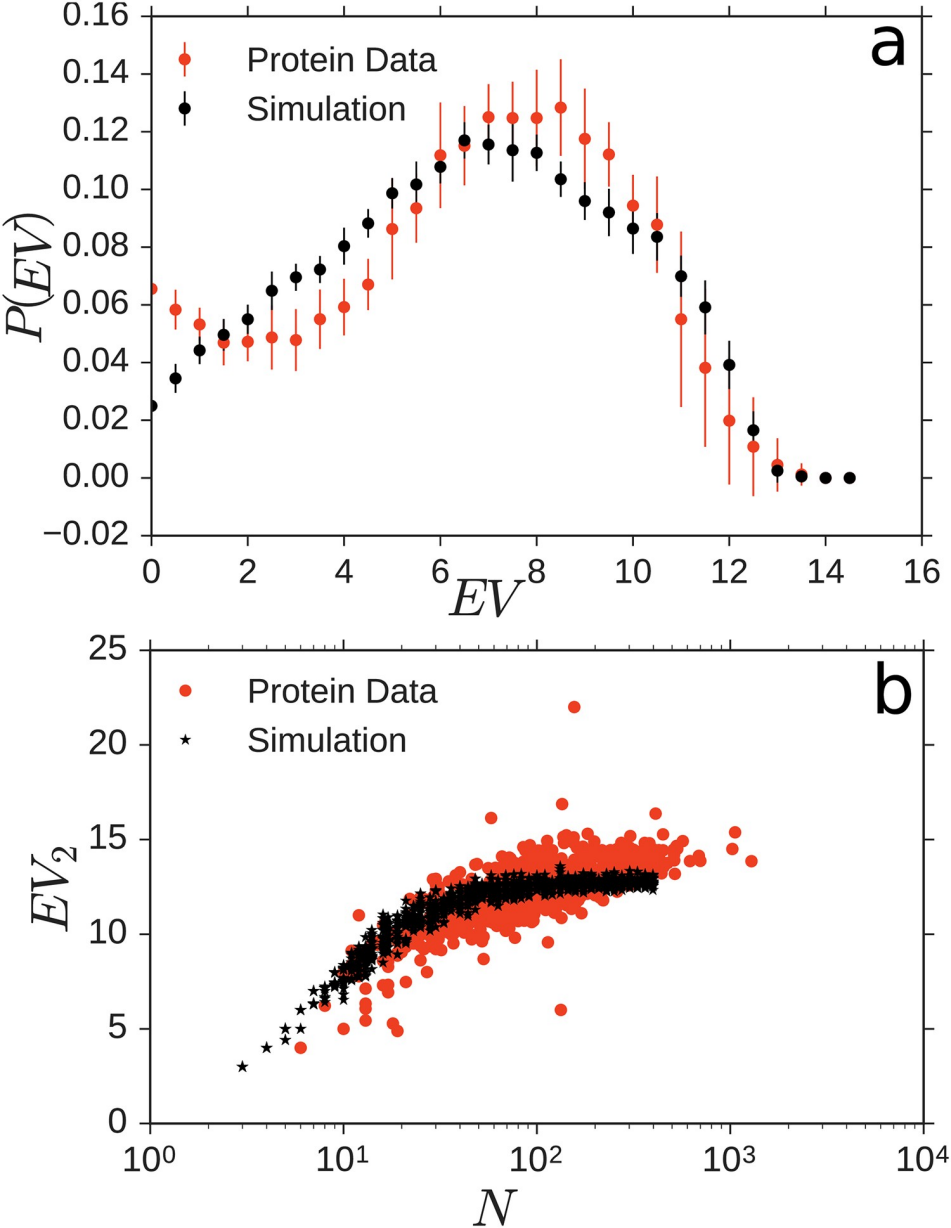
Model eigenvalue spectra of the graph Laplacian are similar to those of the PRN spectra. a) Histograms *P*(*EV*) of eigenvalue spectra of PRN’s with *N* ≈ 400 and *r*_*c*_ = 6.5 Å compared to model output at *N* = 400. b) Second largest eigenvalues *EV*_2_ grow in similar ways for simulation and data. All eigenvalues *λ*_1_, …, *λ*_*N*_ for an (*NxN*) Laplacian matrix *L*^*s*^ *im* = *A*^*s*^ *im* − *diag*_*i*_(*A*_*ii*_) are computed using the routine provided by NetworkX [38]; the second largest eigenvalue *λ*_2_ = *EV*_2_ of those is plotted in panel b.

Lastly we explore the scaling of the second largest eigenvalue of the graph Laplacian with *N* in Fig 6 and find that it grows with *N*, approaching a saturation point of ≈15 for large *N*.

As two additional features roughly characterizing the dynamic properties of protein residue networks, we consider the distribution and scaling of Laplacian eigenvalues. The Laplacian of a network captures both its interaction topology and its relaxation and vibration properties [41, 42]. If the PRN were made only of the central *C*_*α*_ atoms, the Laplacian would exactly quantify the networks vibrational and relaxational modes. As real PRNs are more complex, the Laplacian spectrum can be taken as a proxy for oscillatory and relaxation dynamics.

Because the eigenvalue spectra intrinsically scale with graph size (here: chain length), we have evaluated the spectra of simulated structures and PRNs of lengths of *N* = 400±30. Fig 6a shows the histogram of eigenvalues for the 18 PRNs (red) in that length range, accumulating all *N* eigenvalues for each of the 18 PRNs. For comparison, we computed 28 simulated structures (black), that fall in the same length range.

Both eigenvalue spectra exhibit a characteristic unimodal shape. The simulated structures have a more symmetric, slightly broader spectrum with a peak at *λ* ≈ 7, while the PRN’s have a slightly sharper peak at *λ* ≈ 8 and higher probabilities for very small eigenvalues. Similarly, the second largest Laplacian eigenvalue exhibits the same qualitative scaling with chain length *N* for PRNs and geometric-constraint model. The second largest eigenvalue of a network’s Laplacian quantifies the time scale of its slowest relaxing mode; as such, its scaling with chain length *N* indicates how intrinsic relaxation time scales change due to the aggregates becoming larger.

The spectra and equally the scaling of the second largest eigenvalues are not indistinguishable between model and biological protein data yet overall exhibit similar properties. Whether or not spectra of model ensemble and PRN ensemble actually agree or disagree cannot be concluded without doubt from the data available, both because at (exactly) fixed chain length *N* there typically is no, one, or only very few proteins available in the real protein data set and because the model realizations at fixed *N* yield very similar spectra due to chain homogeneity. There is no unbiased way we know of to account for uncertainties in *N* and simultaneously inhomogeneities in the chain units such that a unambiguous conclusion can be drawn.

## IV. Discussion

In this article we have proposed a simple model of spatial network formation taking into account geometric constraints only. Decoupling the constraints, that drive the folding process (geometry, sequence and solution) and focusing on the geometry allows us insights into the folding mechanisms behind the ensemble features. While this approach does not yield direct predictive power to find the native state of a specific sequence it may narrow down the landscape of possibilities.

We find that geometrically constrained random linking already leads to strong similarities of the resulting structures with protein residue networks in biology. Generalizing a 2D model of purely graph-theoretical network formation presented in [39] to 3D, the model is based upon random link additions with geometric constraints. As the topological shortcut is no longer possible, the geometric constraints are simulated directly. The simulation results were then compared to protein residue networks (PRN’s), choosing the threshold such that the mean degrees of simulation results and PRN’s matched. As a result, the degree distributions are within the error margins of each other.

The network diameter is linearly related to the radius of gyration in both simulation and data and matches when the simulation results are correctly scaled with the mean connection lengths. The network diameter scales with the chain length as a shifted power law with an exponent of *ν*_*sim*_ = 0.345±0.01, which is in agreement with value of *ν*_*exp*_ = 0.374±0.03, measured in PRN’s. As in 2D, this is slightly less than space filling.

Furthermore, we have studied the Laplacian eigenvalue spectrum and the scaling of the second largest eigenvalue with system size, finding that the two systems are compatible. Using the findings from [41, 42] we can infer that the structure of vibrational modes and relaxation properties produced by the model are similar to those found in biological proteins.

These results can be taken as an indication that geometric constraints may be a mechanism behind the scaling behaviour of real protein structures, generating an ensemble also compatible on degree distribution and Laplacian spectrum. Further research, however, is necessary to determine how far the structural similarity reaches. For example by comparing further topological characteristics of PRN’s vs. model simulations. If the analogy persists, the model could be extended to allow simple sequence features, such as hydrophobicity to attempt to get a simpler predictive model. This may give insights into the folding process, that are otherwise lost in simulation complexity.

Taken together, the above results indicate that coarse ensemble properties of protein tertiary structures are already induced by geometric constraints alone such that only finer scales of the folded structures of individual proteins may be controlled by the details of their amino acid sequences. Such simple models provide a new angle of analyzing protein structures at the coarse scale of ensembles and may help understand core mechanisms underlying the complex folding processes.

## Supporting information

S1 File (PDF)
