# Supplementary material for: Self-organized emergence of folded protein-like network structures from geometric constraints
Source: PLoS One. 2020 Feb 27;15(2):e0229230. doi: 10.1371/journal.pone.0229230 (PMC7046222; doi:10.1371/journal.pone.0229230)
Supplement: S1 File — (PDF) [file pone.0229230.s001.pdf]

Supplementary Information accompanying "Geometric constraints in protein folding"

Nora Molkenthin

Network Dynamics, Max Planck Institute for Dynamics and Self-Organization (MPIDS), 37077 Göttingen, Germany

Steffen Mühle

University of Göttingen, Third Institute of Physics – Biophysics, Friedrich-Hund-Platz 1, 37077 Göttingen, Germany.

Antonia S. J. S. Mey

EaStCHEM School of Chemistry, University of Edinburgh, Edinburgh, United Kingdom

Marc Timme

Chair for Network Dynamics, Institute for Theoretical Physics and Center for Advancing Electronics Dresden (cfaed), Technical University of Dresden, 01069 Dresden and Network Dynamics, Max Planck Institute for Dynamics and Self-Organization (MPIDS), 37077 Göttingen, Germany

(Dated: July 4, 2019)

A. Simulation method of the geometric constraint protein model

We have simulated the process modifying the chain geometry in 3D and tested the geometric constraints according to an algorithm consisting of repeated cycles of:

1. A pair (i^*, j^*) of non-adjacent spheres is randomly chosen from the uniform distribution among the set of untried pairs.

2. The two spheres are attempted to be connected by switching on a force of unit strength pointing towards each other (see Fig. 2 of the main article), under the geometric constraints:
   (i) the backbone spheres stay together
   (ii) no spheres overlap
   (iii) spheres connected previously stay together.

3. If the selected spheres touch, a new link between them forms and we update the adjacency matrix by setting the elements $A^{\text{sim}}_{i^*j^*} = A^{\text{sim}}_{j^*i^*} = 1$. Alternatively, if the spheres move less than a velocity threshold $\Delta R/\Delta t$, the link is discarded and marked geometrically impossible (see below for details).

This process is repeated until no further link remains untried. During each cycle, to emulate the direct motion of spheres towards each other and to continuously match all geometric constraints, we change the spheres' positions x_i, $i \in \{1, \dots, N\}$, according to simple overdamped dynamics

$$\mathrm{d}x_i/\mathrm{d}t = \zeta F_i(x),$$

where $x = (x_1, \dots, x_N)^\mathsf{T}$ is the collection of all positions and $F_i(x)$ is the sum of all constraint forces acting on sphere i and, if $i \in \{i^*, j^*\}$, the unit force of magnitude 1. The space and time scale were chosen such that all quantities are dimensionless, the single-sphere friction coefficient ζ is set to 1 and a distance of $x = 1$ corresponds to a bond length, whose mean for real proteins equals 5.066 Å.

The constraints are approximated by taking the total force

$$F_i(x) = -\nabla_i V(x) + F_i^{\mathsf{connect}}(x), \tag{1}$$

as the sum of the forces inducing the connection attempt as

$$F_i^{\mathsf{connect}}(x) = (\delta_{i,i^*} - \delta_{i,j^*}) \frac{x_{j^*} - x_{i^*}}{\|x_{j^*} - x_{i^*}\|}. \tag{2}$$

and the constraint forces that are gradients of summed potentials

$$V(x) = \frac{K}{2} \sum_{n,m=1}^{N} \frac{1}{2} (d_{n,m} - 1)^2 \big(A^{\mathsf{sim}}_{nm} + \Theta(1 - d_{n,m}) \big) \tag{3}$$

quadratic in the distances $d_{n,m} = \|x_n - x_m\|$. Here the Heaviside step function is defined as $\Theta(y) = 0$ if $y < 0$ and $\Theta(y) = 1$ if $y \geq 0$. The first term in the final parenthesis in (3) ensures keeping neighboring units along the chain in contact as well as all other pairs of spheres linked so far during the process. The second term causes overlapping spheres to repel each other. K is an elastic constant chosen large enough for the constraints to be virtually fulfilled and the final chain statistics being invariant of choosing larger values for K, but small enough in order not to limit the allowed numerical time steps unnecessarily. The value $K = 50$ was found to meet these conditions and is used in simulations throughout the article.

The initial configuration of the chain was drawn from a Boltzmann distribution with probability $p = Z^{-1} \exp(-E_{\mathrm{Bend}}/k_B T)$ with $k_B T = 1$ and energy

$$E_{\mathrm{Bend}} = -\kappa \sum_{n=2}^{N-1} \cos(\theta_n), \tag{4}$$

where Z is a normalization constant and θ_n is the bending angle at the nth unit of the chain, defined as the angle between the adjacent tangential vectors through the scalar (dot) product $\cos\theta_n = (x_n - x_{n-1}) \cdot (x_{n+1} - x_n)$, noting that the sphere diameter equals unity. Initially, generated chains were rejected if any constraint was violated. The prefactor κ can be interpreted as bending stiffness and determines the persistence length of the initial chains. It was set to $\kappa = 5$ such that initial chains are slightly bent (See Fig. 2 of the main article for an example).

During a cycle started by selecting the spheres i^* and j^* to be pulled together, we monitored their decreasing distance d_{i^*,j^*}. As soon as $d_{i^*,j^*} \leq 1$, the cycle is considered successful and a new link is formed. We have also periodically checked at intervals ΔT whether d_{i^*,j^*} has shrunk by less than a threshold distance $\Delta R = \Delta T \times \chi \times 2/(N/2)$. If this is the case, the cycle is discarded as unsuccessful, because the pair of units cannot make contact due to geometric constraints. The configuration at the beginning of that cycle is then restored. The last factor in ΔR is the relative velocity of the spheres i^* and j^* in case both – in order to move – have to drag half the other spheres $(N/2)$ along. This lower velocity threshold

was further decreased by introducing the factor $\chi = 0.3$ because the final chain statistics weakly varied for larger values but remained the same for smaller values. We have found $\Delta T = 0.15$ to be small enough in order not to waste computational time on unsuccessful cycles, but large enough to not abort cycles in which d_{i^*,j^*} shrinks slowly only temporarily.

The excluded volume forces are nonzero only for pairs of spheres whose distance is less than one. To speed up the simulation, they were only evaluated for spheres that are elements of each others *neighbor list* listing all spheres within a distance $1 + \epsilon$. We initially generated these lists, then integrated the maximum velocity of all spheres over time and updated the neighbor lists whenever the resulting value exceeded ϵ. The value $\epsilon = 0.2$ provided the best speed-up. At each integer multiple of 100 cycles, all untried links to a sphere i with $\sum_{j=1}^{N} A_{ij}^{\mathsf{sim}} =: k_i \geq 12$ were discarded. This measure was taken to accelerate the simulations as further bonding trials including this sphere are geometrically impossible.

B. Protein Database protein structure preparation

From reference [1] we obtained the list of PDB files used for their analysis. We split the list into NMR structures and X-ray crystal structures, as the NMR structures would contain multiple protein configurations in their PDB entry. Each PDB was then processed with a custom python script that would count the number of C-α atoms found in the structure and order the PDB IDs according to the length of the protein chain. Then from this ordered list every 10th protein was picked, ensuring a good spread of length distributions, a good sample size while also keeping computations easily doable on a workstation. For NMR structures the first structure in the PDB entry was chosen. C-α coordinates were then extracted for each protein using MDAnalysis [2, 3], from which protein residue contact networks were computed using a cutoff distance of $d_c = 6.5$ Å. This allows the comparison of the computationally generated adjacency matrix to the PRN generated one. For the network measures and manipulations NetworkX [4] was used.

All simulation details, including the code for reproducing the geometric constraint simulations, as well as the preparation and analysis of PDB files can be found in the following github

repository: `https://github.com/ppxasjsm/Geometric-constraints-protein-folding`

---

[1] Liu Hong and Jinzhi Lei. Scaling law for the radius of gyration of proteins and its dependence on hydrophobicity. *Journal of Polymer Science, Part B: Polymer Physics*, 47(2):207–214, 2009.

[2] Naveen Michaud-Agrawal, Elizabeth J. Denning, Thomas B. Woolf, and Oliver Beckstein. Mdanalysis: A toolkit for the analysis of molecular dynamics simulations. *Journal of Computational Chemistry*, 32(10):2319–2327, 2011.

[3] Richard J. Gowers, Max Linke, Jonathan Barnoud, Tyler J. E. Reddy, Manuel N. Melo, Sean L. Seyler, Jan Domański, David L. Dotson, Sébastien Buchoux, Ian M. Kenney, and Oliver Beckstein. MDAnalysis: A Python Package for the Rapid Analysis of Molecular Dynamics Simulations. In *Proceedings of the 15th Python in Science Conference*, pages 98 – 105, 2016.

[4] Aric A. Hagberg, Daniel A. Schult, and Pieter J. Swart. Exploring network structure, dynamics, and function using networkx. In *Proceedings of the 7th Python in Science Conference*, pages 11 – 15, 2008.
